# Supplementary figures and images for: Divulging diazotrophic bacterial community structure in Kuwait desert ecosystems and their N2-fixation potential
Source: PLoS One. 2019 Dec 26;14(12):e0220679. doi: 10.1371/journal.pone.0220679 (PMC6932743; doi:10.1371/journal.pone.0220679)

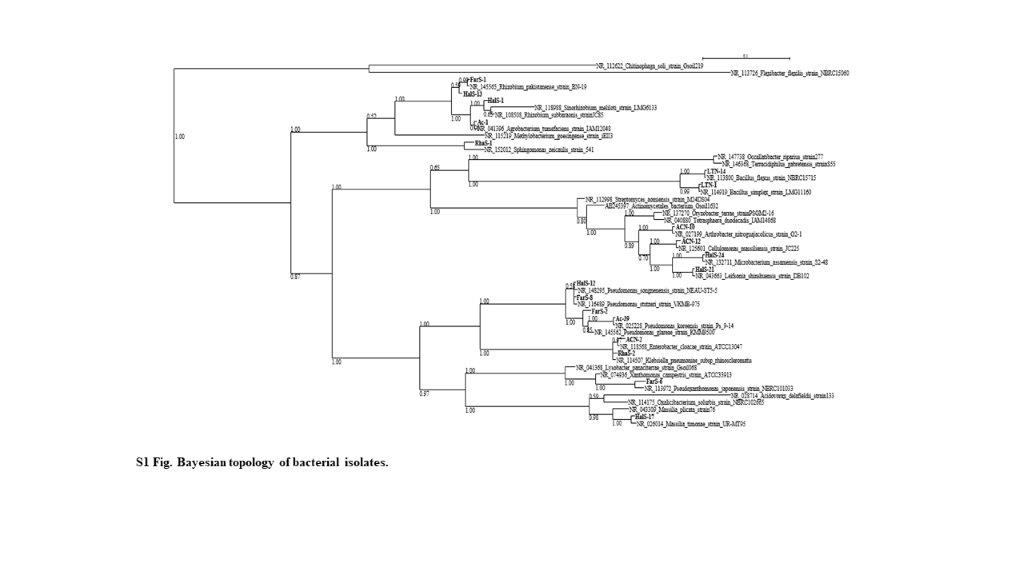

Supplement: S1 Fig — (JPG) [file pone.0220679.s001.jpg]

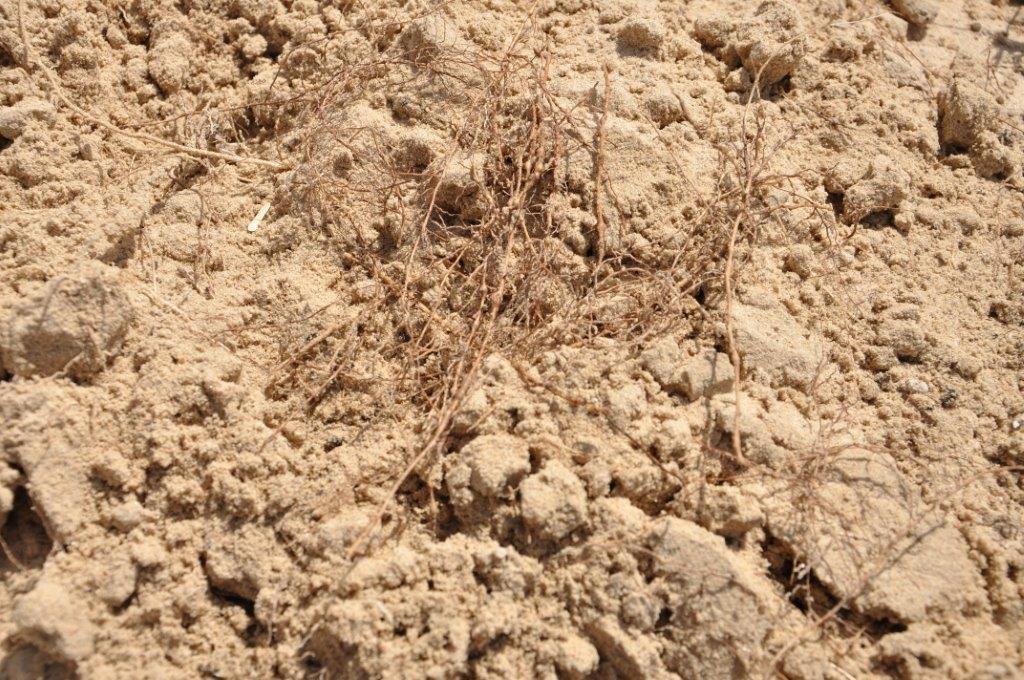

Supplement: S2 Fig — (JPG) [file pone.0220679.s002.jpg]
