# Supplementary material for: Divulging diazotrophic bacterial community structure in Kuwait desert ecosystems and their N2-fixation potential
Source: PLoS One. 2019 Dec 26;14(12):e0220679. doi: 10.1371/journal.pone.0220679 (PMC6932743; doi:10.1371/journal.pone.0220679)
Supplement: Attachment — Submitted filename: Abstract.docx [file pone.0220679.s004.docx]

**Abstract**

Kuwait is a semi-arid region with soils that are relatively nitrogen-poor . Thus, biological nitrogen fixation is an important natural process in which N2-fixing bacteria (diazotrophs) convert atmospheric nitrogen into plant-usable forms such as ammonium and nitrate. Currently, there is limited information on free-living and root-associated nitrogen-fixing bacteria and their potential to fix nitrogen and aid natural plant communities in the Kuwait desert. In this study, free living N2-fixing diazotrophs were enriched and isolated from the rhizosphere soil associated with three native keystone plant species; *Rhanterium epapposum*, *Farsetia* *aegyptia*, and *Haloxylon salicornicum*. Root-associated bacteria were isolated from the root nodules of *Vachellia pachyceras*. The result showed that the strains were clustered in five groups including the Actinobacteria being the most dominant phyla followed by Firmicutes, and the classes β-proteobacteria γ-proteobacteria, and α-proteobacteriaThis study initially identified 50 nitrogen-fixers by16S rRNA gene sequencing, of which 78% were confirmed to be nitrogen-fixers using the acetylene reduction assay. Among the nitrogen fixers identified, the genus *Rhizobium* was predominant in the rhizosphere soil of *R. epapposum* and *H.* *salicornicum*, whereas *Pseudomonas* was predominant in the rhizosphere soil of *F. aegyptia*, . The species *Agrobacterium tumefaciens* was dominant in the root nodules of *V. pachyceras*. These results indicate that plant species and their rhizosphere effects are important drivers of diazotroph diversity in arid soils. To our knowledge, this study is the first to use culture-based isolation, molecular identification, and evaluation of N2-fixing ability to detail diazotroph diversity in Kuwaiti desert soils.
